# Projected early spread of COVID-19 in Africa through 1 June 2020

**DOI:** 10.2807/1560-7917.ES.2020.25.18.2000543

**Published:** 2020-05-07

**Authors:** Carl AB Pearson, Cari Van Schalkwyk, Anna M Foss, Kathleen M O’Reilly, SACEMA Modelling and Analysis Response Team, CMMID COVID-19 working group, Juliet RC Pulliam

**Affiliations:** 1 London School of Hygiene & Tropical Medicine, London, United Kingdom; 2 South African DSI-NRF Centre of Excellence in Epidemiological Modelling and Analysis (SACEMA), Stellenbosch University, Stellenbosch, Republic of South Africa; 3 Members of the SACEMA Modelling and Analysis Response Team are listed at the end of this article; 4 Members of the CMMID COVID-19 working group are listed at the end of this article

**Keywords:** Africa, COVID-19, computer simulation, epidemics

## Abstract

For 45 African countries/territories already reporting COVID-19 cases before 23 March 2020, we estimate the dates of reporting 1,000 and 10,000 cases. Assuming early epidemic trends without interventions, all 45 were likely to exceed 1,000 confirmed cases by the end of April 2020, with most exceeding 10,000 a few weeks later.

The World Health Organization (WHO) declared coronavirus disease (COVID-19) a public health emergency of international concern on 30 January 2020 [1] and then a pandemic on 11 March 2020 [2], highlighting its rapid global spread and risk of overwhelming healthcare services with patients requiring critical care. By 22 April 2020, WHO situation reports (SITREPs), indicated 56 African countries/territories reporting at least one laboratory-confirmed infection (‘reported case’) of COVID-19 [3]. We aim to estimate the timing of when countries/territories with early cases (reported before 10:00 Central European Summer Time (CEST) 23 March 2020, 45 of those 56) would report 1,000 and 10,000 cases [4].

## Assumptions, data sources and model used in the study

Reported cases underestimate actual infections due to the mix of those resulting in mild disease and asymptomatic infections [5-7], the similarity of symptoms to other diseases common to lower-middle income countries [8], and the limitations of local surveillance systems [9]. However, assuming that the reporting fraction among actual cases and delay are constant, reported cases grow in proportion to the underlying epidemic, thus even with under-ascertainment of the number of actual cases, reported cases provide a useful predictor for stress on healthcare systems. We can use this surrogate for the real epidemic to forecast future trends, and understand what preparations need to be made now and the consequences of slow public health responses.

We employed a branching process model to project the number of future cases of COVID-19 in each country/territory. This model assumes each case produces a number of new cases (distributed *N* ∼ NegBinom(*R* = 2, *k* = 0.58), using the reproductive number* R* estimate from [10] and the dispersion* k* estimate from [11]) occurring after some serial interval (distributed *T* ∼ LogNormal(E[*X*] = 4.7, SD[*X*] = 2.9, in days, with the distribution characterised by the expected value (E) and standard deviation (SD)). We also considered *R* = 3 as a sensitivity study given consensus that the reproductive number has been higher outside of Africa [12]. We start with cases timed according to the first 25 (or fewer) reported cases in the WHO SITREPs up to 23 March 2020 (SITREP 63) [4]. Using those epidemic parameters and initial cases and dates, we simulate the accumulation of reported cases. We assume there are always sufficient unreported infections to continue transmission, and that new cases represent a reporting sample from both identified and previously unidentified transmission chains. As long as a constant reporting fraction and delay persists during this period, and unreported spread is large relative to reported cases (or reporting does not represent effective control), this is a reasonable approximation.

For each set of country-specific initial conditions, we generate n = 10,000 epidemics, discarding any that fade out, consistent with our assumption of unreported transmission chains. We identify the dates when each simulation run crosses 1,000 and 10,000 reported cases, and then evaluate the 50% and 95% quantiles of those dates to determine the forecast interval. The model was built in the R statistical programming language, using the *bpmodels* package [13], and the *data2019nCoV* package for the SITREP data [14]. All analysis code is available at https://github.com/SACEMA/COVID10k.

## Projected timing of 1,000 and 10,000 COVID-19 case reporting in considered African countries/territories 

**Figure 1:**
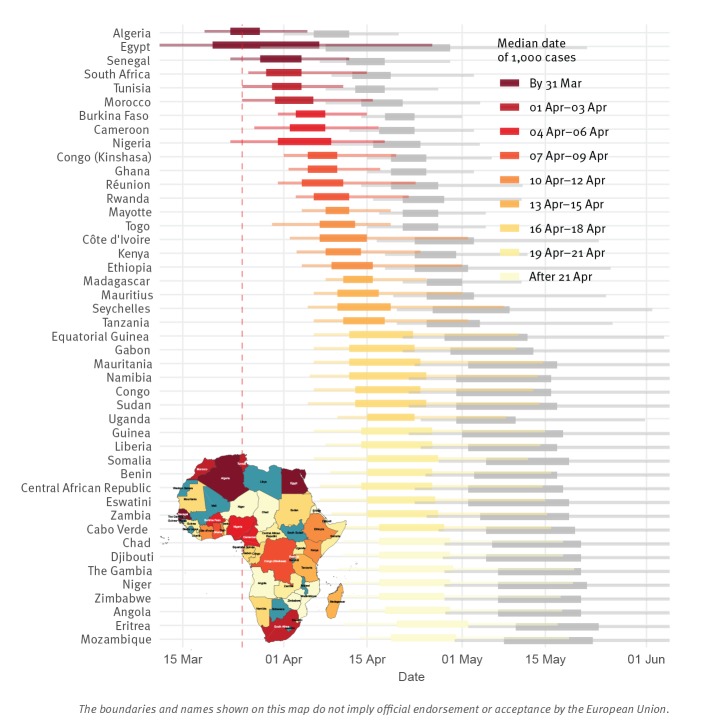
Distribution of times when countries/territories already reporting COVID-19 cases by 23 March 2020 arrive at 1,000 and 10,000 reported cases, with inset map indicating median expected arrival dates by country/territory, Africa, 2020 (n = 45 countries/territories)

We projected that all of the 45 African countries reporting cases prior to 23 March 2020 were likely to pass 1,000 reported cases by the end of April 2020 and 10,000 within another few weeks (Figure 1 and Table). Of particular concern is that passing these thresholds is largely synchronised continent-wide and real disease burden will certainly exceed reported cases. Assuming *R =* 3 instead, reporting of 1,000 cases occurs roughly 1 week earlier, and 10,000 cases 2 weeks earlier. However, early trends in cases for the *R* = 3 scenario rapidly exceeded observed reporting, indicating epidemiological differences; whether those distinctions are in transmission or surveillance and response remains to be determined.

For each country, the respective predicted dates of reaching 1,000 and 10,000 reported cases are each within coloured (dark red to yellow) and grey horizontal boxes, representing the 50% quantiles for these dates (i.e. 50% forecast intervals). The whiskers surrounding the boxes are 95% forecast intervals.

For the predicted periods of arrival at 1,000 reported cases, the horizontal boxes and whiskers are coloured according to a gradient from dark red to yellow. The dark red progresses to yellow as the forecast intervals of arrival to 1,000 reported cases are delayed in time.

The dashed vertical line indicates cut-off date for initial cases in the simulated epidemics.

The inset map indicates the median expected arrival dates at 1,000 reported cases by country/territory (dark red to yellow, corresponding to the forecast distributions); countries/territories not reporting cases by 23 March 2020 SITREP are in blue.

**Table:** Projected timing of reporting of 1,000 and 10,000 COVID-19 cases for all African countries/territories reporting cases by 23 March 2020, assuming no change in reporting or introduction of control efforts, Africa, 2020 (n = 45 countries/territories)

Country/territory	Forecast intervals 50% (95%) around date of 1,000 reported cases	Forecast intervals 50% (95%) around date of 10,000 reported cases	Date SI > 30
Algeria	23 Mar–28 Mar (18 Mar–05 Apr)	06 Apr–12 Apr (01 Apr–20 Apr)	12 Mar
Angola	18 Apr–29 Apr (12 Apr–17 May)	07 May–21 May (28 Apr–12 Jun)	24 Mar
Benin	15 Apr–26 Apr (08 Apr–16 May)	03 May–17 May (24 Apr–09 Jun)	19 Mar^a^
Burkina Faso	03 Apr–08 Apr (31 Mar–15 Apr)	18 Apr–23 Apr (14 Apr–01 May)	16 Mar
Cabo Verde	17 Apr–28 Apr (11 Apr–16 May)	05 May–20 May (27 Apr–11 Jun)	13 Mar^a^
Cameroon	02 Apr–08 Apr (27 Mar–17 Apr)	17 Apr–23 Apr (12 Apr–03 May)	27 Mar
Central African Republic	14 Apr–26 Apr (07 Apr–14 May)	02 May–18 May (23 Apr–09 Jun)	18 Mar^a^
Chad	17 Apr–29 Apr (11 Apr–18 May)	06 May–21 May (28 Apr–12 Jun)	26 Mar
Congo	13 Apr–24 Apr (06 Apr–13 May)	30 Apr–16 May (22 Apr–06 Jun)	19 Mar^a^
Côte d’Ivoire	07 Apr–15 Apr (02 Apr–02 May)	23 Apr–03 May (16 Apr–24 May)	18 Mar^a^
Democratic Republic of the Congo	05 Apr–10 Apr (01 Apr–19 Apr)	19 Apr–25 Apr (15 Apr–06 May)	19 Mar
Djibouti	17 Apr–29 Apr (11 Apr–18 May)	05 May–21 May (27 Apr–12 Jun)	19 Mar
Egypt	20 Mar–07 Apr (09 Mar–26 Apr)	08 Apr–29 Apr (28 Mar–22 May)	19 Mar
Equatorial Guinea	12 Apr–22 Apr (06 Apr–10 May)	28 Apr–12 May (21 Apr–04 Jun)	15 Mar^a^
Eritrea	20 Apr–02 May (11 Apr–17 May)	10 May–24 May (02 May–11 Jun)	17 Mar^a^
Eswatini	15 Apr–26 Apr (07 Apr–15 May)	02 May–19 May (23 Apr–10 Jun)	17 Mar
Ethiopia	09 Apr–16 Apr (04 Apr–01 May)	23 Apr–02 May (18 Apr–26 May)	16 Mar^a^
Gabon	12 Apr–23 Apr (06 Apr–10 May)	29 Apr–13 May (21 Apr–05 Jun)	14 Mar
Gambia	17 Apr–29 Apr (10 Apr–19 May)	07 May–21 May (28 Apr–12 Jun)	18 Mar
Ghana	05 Apr–10 Apr (01 Apr–17 Apr)	19 Apr–25 Apr (15 Apr–03 May)	16 Mar
Guinea	14 Apr–26 Apr (06 Apr–15 May)	01 May–18 May (22 Apr–09 Jun)	26 Mar^a^
Kenya	08 Apr–14 Apr (03 Apr–24 Apr)	23 Apr–30 Apr (18 Apr–12 May)	13 Mar
Liberia	14 Apr–26 Apr (08 Apr–14 May)	02 May–17 May (24 Apr–09 Jun)	22 Mar^a^
Madagascar	11 Apr–16 Apr (08 Apr–25 Apr)	25 Apr–01 May (21 Apr–11 May)	21 Mar
Mauritania	12 Apr–24 Apr (06 Apr–14 May)	02 May–17 May (23 Apr–07 Jun)	19 Mar
Mauritius	10 Apr–17 Apr (06 Apr–01 May)	25 Apr–03 May (19 Apr–25 May)	20 Mar
Mayotte	08 Apr–12 Apr (04 Apr–19 Apr)	21 Apr–27 Apr (17 Apr–05 May)	N/A
Morocco	30 Mar–06 Apr (24 Mar–16 Apr)	14 Apr–21 Apr (08 Apr–04 May)	13 Mar
Mozambique	19 Apr–30 Apr (13 Apr–19 May)	08 May–23 May (29 Apr–14 Jun)	24 Mar
Namibia	12 Apr–25 Apr (05 Apr–13 May)	30 Apr–16 May (22 Apr–07 Jun)	17 Mar
Niger	17 Apr–29 Apr (11 Apr–19 May)	07 May–22 May (28 Apr–12 Jun)	19 Mar
Nigeria	31 Mar–09 Apr (23 Mar–18 Apr)	16 Apr–24 Apr (09 Apr–04 May)	18 Mar
Réunion	04 Apr–11 Apr (31 Mar–23 Apr)	19 Apr–27 Apr (14 Apr–11 May)	14 Mar
Rwanda	06 Apr–12 Apr (02 Apr–22 Apr)	21 Apr–28 Apr (16 Apr–11 May)	14 Mar
Senegal	28 Mar–04 Apr (23 Mar–12 Apr)	11 Apr–18 Apr (07 Apr–29 Apr)	15 Mar^a^
Seychelles	10 Apr–19 Apr (05 Apr–08 May)	26 Apr–09 May (20 Apr–02 Jun)	22 Mar
Somalia	15 Apr–27 Apr (06 Apr–12 May)	05 May–19 May (27 Apr–06 Jun)	31 Mar^a^
South Africa	29 Mar–04 Apr (26 Mar–15 Apr)	12 Apr–19 Apr (09 Apr–03 May)	15 Mar
Sudan	13 Apr–25 Apr (05 Apr–14 May)	30 Apr–17 May (22 Apr–08 Jun)	14 Mar
Togo	07 Apr–13 Apr (30 Mar–19 Apr)	21 Apr–27 Apr (15 Apr–05 May)	20 Mar^a^
Tunisia	30 Mar–04 Apr (25 Mar–11 Apr)	13 Apr–18 Apr (08 Apr–27 Apr)	09 Mar
Uganda	15 Apr–23 Apr (10 Apr–08 May)	30 Apr–10 May (24 Apr–31 May)	18 Mar
Tanzania	11 Apr–18 Apr (06 Apr–02 May)	25 Apr–04 May (20 Apr–26 May)	18 Mar
Zambia	15 Apr–27 Apr (09 Apr–15 May)	04 May–19 May (25 Apr–11 Jun)	20 Mar
Zimbabwe	17 Apr–28 Apr (11 Apr–17 May)	07 May–21 May (28 Apr–11 Jun)	23 Mar

COVID-19: coronavirus disease; N/A: data not available; SI: stringency index.

^a^ Denotes countries where the date is estimated by manually coding from [17].

As a general assessment of the validity of assuming limited impact of control measures during the timeframe modelled, the last column denotes the date the SI [18] surpassed 30. The SI is an amalgamation of several intervention policies, which measures their formal declaration, and does not imply anything about their effectiveness.

Since our projections assume failed containment of initial cases and no interventions reducing early transmission, they are pessimistic relative to any benefits of local (e.g. those summarised by the stringency index (SI) in the Table) or global action. The international control efforts and travel restrictions to date likely slowed seeding of the epidemic, and other local efforts may have slowed spread once the epidemic was established. However, containment measures, e.g. travel restrictions, increased testing, contact tracing, isolation of cases and quarantine of contacts, are likely to slow, but not halt, real epidemic growth [15]. Indeed, increased testing may accelerate the time to reporting these numbers, as improved ascertainment increases the identified fraction of real cases. However, the model also optimistically assumes surveillance capacity is not overwhelmed or stymied, which would slow reaching 1,000 reported cases while the real disease burden grows uncontrolled. This model also assumes a homogeneous population, neglecting urban vs rural differences as well as connectivity between regions, distinctions, which would tend towards many small outbreaks, rather than the single one we model. Because we ignore these effects, the model is only appropriate for short-range forecasts.

## Validation of the model

**Figure 2:**
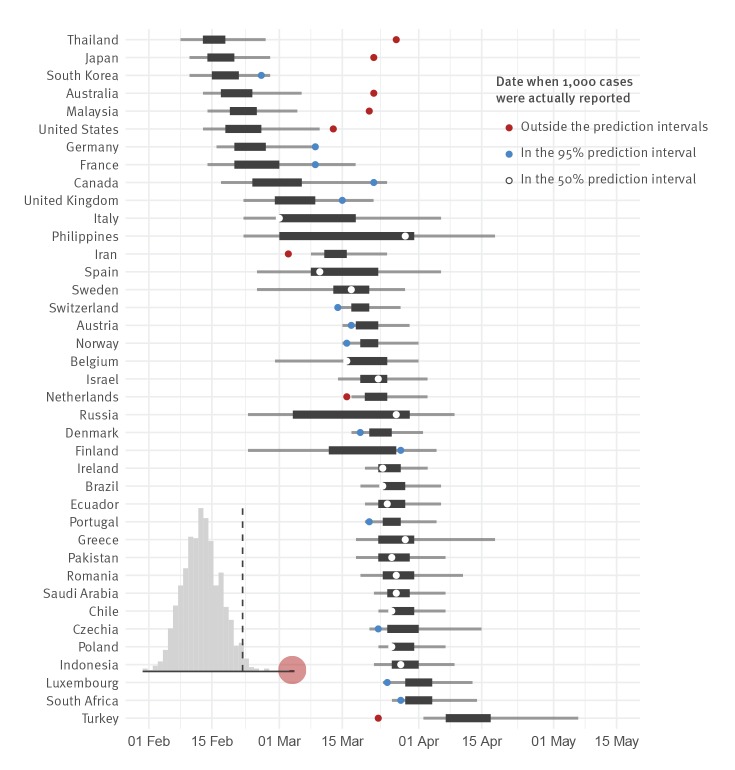
Forecast validation for countries having already reached 1,000 cases, 2020 (n = 39 countries)

As model validation, we applied this same forecasting approach to the 39 countries worldwide that had exceeded 1,000 reported cases by 7 April (SITREP 78) [16]; we did not consider those with more than 10,000 cases, as they all underwent substantial control measures modifying epidemic growth. We found that 17/39 of actual reporting dates fell within the 50% prediction intervals, and 31/39 within the 95% interval (Figure 2), indicating the forecast prediction interval is too certain, as expected for a rapid but low detail model. We further showed that forecast performance is not a random outcome by performing a randomisation test: we shuffle the assignment of forecast days-to-1,000-cases to different countries, and score 1,000 shuffled predictions; the real forecast score is significantly different from random at the p < 0.001 level (Figure 2 inset).

For each country, the predicted date of reaching 1,000 reported cases is within a black horizontal box, representing the 50% quantiles for the date (i.e. 50% forecast interval). The grey whiskers surrounding the boxes are 95% forecast intervals. The dot indicates the actual date when 1,000 reported cases were reached, with white, blue or red colours respectively signalling whether this date falls within the 50% forecast interval, outside the 50% forecast interval but still within the 95% interval, or outside the 95% interval.

For actual reporting dates, 17/39 fell within the 50% prediction intervals, and 31/39 within the 95%.

Inset distribution indicates randomisation results: actual forecast (red) vs randomly assigned forecast (grey), with 0.975 quantile indicated by the line.

Specific to Africa as of SITREP 93 (22 April 2020), Egypt, Morocco, and South Africa actually reached 1,000 reported cases at a date falling within their 50% forecast interval, and Algeria within its 95% interval; Cameroon and Ghana reported 1,000 cases later than their 95% interval. For 18 other countries the upper time limit of the 50% forecast interval had already been reached on 22 April, with 11 still in their 95% interval; one of those had > 900 cases (Côte d’Ivoire). Of the seven now past their 95% interval (Burkina Faso, DR Congo, Nigeria, Rwanda, Senegal, Togo, Tunisia), one has > 900 cases (Tunisia) and the others generally had early interventions (SI column in the Table).

## Conclusions

Using reporting to date, and assuming similar epidemiological trends to those seen globally, we projected that all of the 45 African countries/territories reporting early cases were likely to report 1,000 cases by the end of April 2020, and 10,000 within another few weeks; with reported cases lagging and under-representing actual burden. Even considering reporting delays, timing is largely synchronised continent-wide. Our projections assumed no substantive changes between the initially reported cases and the forecast points; while some countries have taken drastic actions, invalidating those assumptions, others have not or have acted slowly (SI column in the Table). As seen outside Africa, because onset of severe symptoms can be delayed weeks from infection and last several weeks, interventions have limited immediate impact on new hospitalisations or healthcare facility demand, meaning that most of the countries in our projection would be well past 1,000 real cases by the time the effects of interventions started in late March would be observed.

These results call for further preparations across Africa to ready healthcare systems and citizens for the incoming wave of infections leading to COVID-19; many countries are already making these changes, but more may be required. Augmented staffing, personal protective equipment stores and training, general isolation beds and equipped critical care units are all urgently needed. Citizen awareness will also be critical, and officials should continue to promote preventive measures such as physical distancing and regular hand washing.

## SACEMA Modelling and Analysis Response Team (SMART)

Roxanne Beauclair, Elisha B Are, Olatunji O Adetokunboh, Jeremy Bingham, C Marijn Hazelbag, Ivy Kombe, and Joseph B Sempa.

## Centre for Mathematical Modelling of Infectious Disease COVID-19 working group

Emily S Nightingale, Sebastian Funk, Rosalind M Eggo, Joel Hellewell, Adam J Kucharski, Quentin J Leclerc, Nicholas G Davies, Jon C Emery, Stefan Flasche, Nikos I Bosse, Sam Abbott, Megan Auzenbergs, Amy Gimma, Simon R Procter, Rein MGJ Houben, Timothy W Russell, Akira Endo, Charlie Diamond, James D Munday, Gwen Knight, Fiona Yueqian Sun, Yang Liu, Arminder K Deol, Thibaut Jombart, Billy J Quilty, Samuel Clifford, Petra Klepac, Kevin van Zandvoort, Kiesha Prem, Alicia Rosello, Graham Medley, Mark Jit, Christopher I Jarvis, Hamish Gibbs, and W John Edmunds.
